# Comparative Analysis of User Exposure to the Electromagnetic Radiation Emitted by the Fourth and Fifth Generations of Wi-Fi Communication Devices

**DOI:** 10.3390/ijerph17238837

**Published:** 2020-11-27

**Authors:** Annamaria Sârbu, Simona Miclăuș, Angela Digulescu, Paul Bechet

**Affiliations:** 1 Communications, IT and Cyber Defense Department, “Nicolae Bălcescu” Land Forces Academy, 550170 Sibiu, Romania; simo.miclaus@gmail.com (S.M.); pbechet@gmail.com (P.B.); 2 Telecommunications and Information Technology Department, “Ferdinand I” Military Technical Academy, 050141 Bucharest, Romania; angela.digulescu@mta.ro

**Keywords:** radiofrequency exposure, Wi-Fi mobile device, amplitude probability density, modulation, incident energy density per bit

## Abstract

A suitable metric to describe human exposure to microwaves emitted by wireless communication devices is still incomplete. By using both theoretical analysis and experimental validation (in controlled and real deployed networks), we analyze and compare the specificity of exposure due to data transmissions in different configurations of fourth and fifth generation wireless fidelity (Wi-Fi) standards in the proximity of a mobile device. Measurements made use of the capability of the amplitude probability density incorporated in a real-time spectrum analyzer, proving its agility of highlighting different user exposure profiles. The results are presented comparatively and indicate that, in Wi-Fi networks, the modulation and coding scheme (MCS) should be used together with the duty cycle for an improved exposure assessment. The present work introduces the emitted energy density per bit in describing the user’s exposure to Wi-Fi signals and proves its superiority in characterizing the true levels of exposure for the IEEE 802.11n and 802.11ac standards of communication.

## 1. Introduction

The wireless fidelity (Wi-Fi) communication protocols started to develop more than two decades ago and since then they have been widely used in both professional and residential networks. Nowadays most of the Wi-Fi devices provide dual band support for communication at both 2.4 GHz (IEEE protocols 802.11 b/g/n [1]) and 5GHz (IEEE protocols 802.11 n/ac/ax [2]) frequency ranges. However, the use of 2.4 GHz bandwidth has reached its data rate limits and has become congested. The least crowded 5 GHz Wi-Fi band offers higher data transfer rates at the cost of a reduced coverage area. It provides a better support for more resource-consuming applications like video streaming. Often, 2.4 GHz and 5 GHz Wi-Fi networks coexist and the users need to choose between them based mainly on the necessary transfer rates or coverage without considering their radiofrequency (RF) exposure. 

Human protection standards related to the exposure to RF radiation have recently been updated [3,4], but the guidelines have been elaborated solely on the basis of unanimously recognized thermal effects on tissues. However, the specialized literature provides numerous studies indicating non-thermal effects of Wi-Fi signals on different biological structures. For example, in [5], authors report increased bacterial resistance to antibiotics due to 2.4 GHz radiation, while in [6] and [7] exposure to Wi-Fi radiation is associated with oxidative stress in the brain and liver of rats. A wide range of papers are available on the subject, but most of the experiments are conducted on animals [6,7,8,9,10,11] or microorganisms [5,12]. The limited available human studies like [13,14] should be interpreted taking into consideration their technical limitations, such as the simple proximity to Wi-Fi routers/clients. Nevertheless, these data are not sufficient to be reported, both because of the very uneven radiation distribution in space and because of the time-variable nature of the transmissions [15]. A 2013 review summarizing the possible health effects of Wi-Fi is presented in [15]. Consequently, knowledge on RF exposure of individuals to Wi-Fi is still incomplete, suggesting the need for future research on this topic. A more recent survey on the subject (2018) reported seven Wi-Fi bioeffects possibly caused by other similar electromagnetic field (EMF) exposures [16]. However, the findings presented in [16] were accused of errors, biased opinion, and methodological drawbacks, as the analyzed studies lacked critical review [17,18,19].

Wi-Fi is one of the main RF technologies found in indoor environments like offices, educational or residential buildings [20]. With particular attention being given to sensitive environments like public schools and hospitals, in the public literature we can find several such exposure studies [21,22,23]. In [21], authors perform a comparative analysis of the results obtained from two different EMF measurement campaigns conducted in 2012 and 2018, respectively. Within a 6-year time span, the value of the cumulative electric field strength increased 3 times in most of the measurement locations [21]. Between these two campaigns, the emergence of the new base stations, as well as numerous Wi-Fi networks, was noticed in the campus. Measurements performed in [22,23] were based on data collected by personal RF exposimeters worn by teachers [22] or placed in different locations on the campus [23]. None of the articles indicated the exceeding of the limits specified by the International Commission on Non-Ionizing Radiation Protection (ICNIRP) guidelines, but exposure was found to vary with the access point positioning, with the wireless application that was used or with the traffic direction [22]. Even if it is very convenient and usually easy to perform, the use of personal RF exposimeters for exposure assessment is subject to several uncertainties as reported in [24,25]. 

Wi-Fi exposure studies like [26], which present detailed description of the exposure system and dosimetry, are scarce. In addition, the majority of the studies focus on measuring exposure in the proximity of the router/access point [26] or on mapping the overall Wi-Fi band exposure within a selected area [23,27] without considering the number of subscribers, the applications that are used, the traffic direction or the Wi-Fi networks settings. 

In this context, the dose-based approach is gaining increased attention in the exposure evaluation studies, since the stochastic-like nature of these signals is continuously evolving. Recent results suggest that, in the fifth generation (5G) networks, the highest dose is always dominated by the individual’s mobile phone and, in the case of non-users, by the bystanders’ mobile phones [28]. This mitigates for exposure evaluation near the mobile device as it is the constant and the closest source of EMF. 

Practically, until 2004, just a single paper discussed the role of modulation on the exposure level and its effects on humans [29]. To our knowledge, in the case of Wi-Fi networks, only the following publications mention that modulation could be important when reporting the user’s exposure, but none provide detailed evidence on the matter [30,31]. Therefore, we consider that a more in-depth analysis would fill that gap and demonstrate if modulation indeed plays a significant role in defining the user’s exposure, for example when Wi-Fi network signals are radiated. 

Based on the previously highlighted aspects retrieved from the literature survey and following some of our previous work [32,33], this article aims to underline differences in terms of user’s exposure to 802.11n (2.4 GHz) and 802.11ac (5 GHz) Wi-Fi signals near a mobile phone—device under test (DUT), based on the measurements performed under different received signal strength conditions, different channel bandwidths, and traffic directions (upload/download) respectively. Authors found no such information in the analysis of the state-of-the-art literature and consider it extremely important as it may represent a milestone in developing exposure profiles, extremely valuable for future epidemiological studies.

To enhance the validity of the experimental results, a second controlled measurement setup was also designed. In this second analysis, the emphasis lays on underlying modulation as a specific dosimetric indicator, providing insight based on both theoretical and experimental results. 

All measurements were performed by using the amplitude probability distribution (APD) capability of a real time spectrum analyzer (RTSA). This statistic capability proves to be agile in capturing the differences inside the exposure profiles due to non-stationary/rapid varying signals and correctly quantifies the true exposure level of the user in different situations.

The remainder of this paper is divided into the following sections and subsections: 2. Materials and Methods; with subsections 2.1 High Fidelity Measurement of the Wi-Fi Signal Levels in Air, and 2.2 Modulation Role on the User’s Exposure Level in IEEE 802.11n and 802.11ac Networks; 3. Results: Indicators of the Human Exposures in the Configured Experimental Scenarios; 4. Modulation: A Significant Indicator of the User’s Exposure in 802.11n and 802.11ac Networks; 5. Discussion; and 6. Conclusions. 

## 2. Materials and Methods

### 2.1. High Fidelity Measurement of the Wi-Fi Signal Levels in Air 

Four Wi-Fi networks of the fourth (IEEE 802.11n) and fifth (IEEE 802.11ac) generations, respectively, were deployed sequentially indoors by means of a TP Link Archer C6 type of router, which is a dual-band wireless router equipped with four fixed external antennas and one internal antenna. The effective isotropic radiated powers (EIRP) at the transmission antennas were: <20 dBm (at 2.4 GHz) and <23 dBm (at 5 GHz). Table 1 presents the configuration of the experimental Wi-Fi networks. Two channel bandwidths were enabled per each of the two Wi-Fi protocols, resulting in four network configurations.

**Figure 1:**
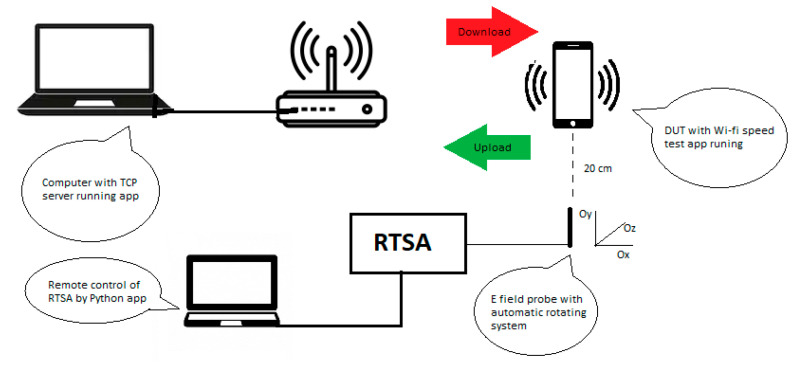
Wi-Fi network components and the measurement flow for determining the exposure level.

A Huawei P10 Lite mobile phone was connected to each of the networks indicated din Table 1. According to Federal Communications Commission (FCC) specifications [34], the conducted power in the Wi-Fi antenna of the mobile phone was of 16 dBm for the 802.11n and of 10 dBm for the 802.11ac standard, respectively. The Wi-Fi networks, composed of the Wi-Fi connected mobile phone and the local Transmission Control Protocol (TCP) server (wired connected to the router), were configured according to Figure 1. A free Android application [35] was used to enable Wi-Fi operation of the DUT. The application performed a local network test between the two devices using the TCP or User Dtagram Protocol (UDP) protocol. The user set the IP address of the remote server (a wired connected computer) and performed limitless upload/download transfers, while measuring the upload, respectively the download throughput. The application enabled the data size and message block size settings. In our experiments, we used limitless data size and a 32768 bytes message block size. Mobile data, Bluetooth, and near field communication (NFC) were disabled at the DUT during measurements. In addition, the Wi-Fi networks were not connected to the internet, thus the network traffic was generated solely by the application, without any background data-consuming applications running. 

**Figure 2:**
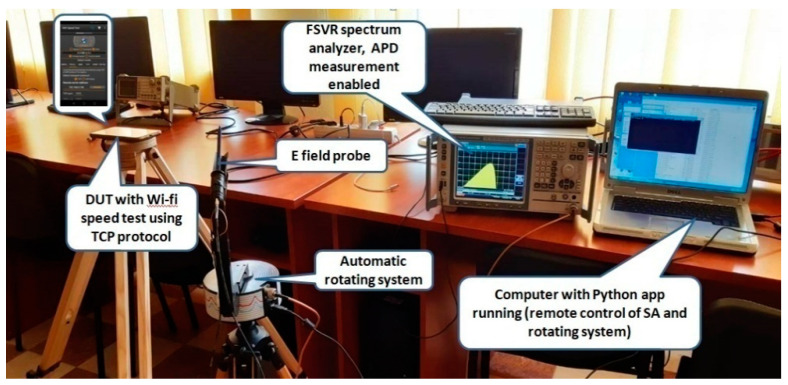
Experimental Wi-Fi measurement setup.

The electric (E) field level was measured by means of a RF near field probe (E-field sniffer), from Aaronia AG, connected to a RTSA -model FSVR by Rohde & Schwarz (R&S). The E field was acquired in a single spatial point in air, situated at a distance of 20 cm from the bottom of the mobile phone (specification of the Wi-Fi antenna position according to the manufacturer). During each measurement, the DUT performed data upload/download by means of the Android application. The field probe was attached to an automatic positioning system to perform three-axis measurements of the E-field strength sequentially. The experimental set-up is shown in Figure 2. The measurements were controlled by a custom-made Python application that enabled the remote controlling of the RTSA, the change of the direction of the probe, and the automatic retrieval of data files. Based on the dimension of the receiving antenna (6 cm) and on the operating wavelengths (12.41 cm and 5.7 cm, respectively) the EMF was measured in far field conditions. The 20 cm distance from the DUT was chosen to determine the field strength (V/m) because we observed that this was the average distance between the DUT and the body (chest) of the user during the period when the file upload/download actions were performed. 

**Figure 3:**
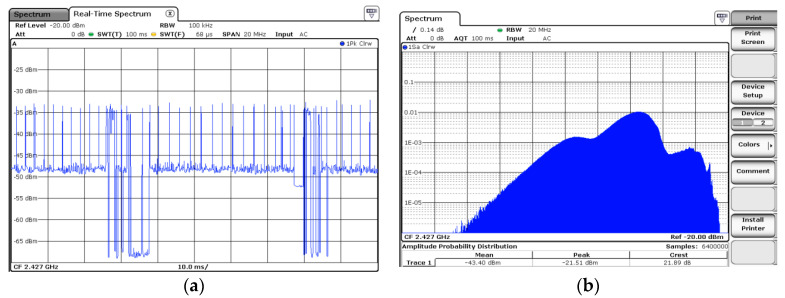
(**a**) Power versus time representation during file upload of a 4th generation Wi-Fi signal, 20 MHz channel bandwidth; (**b**) APD representation of the same signal, at AQT = 100 ms.

The positions of the DUT and that of the measurement system were kept unchanged during the measurements, but the wireless router was positioned in a set of 9 different locations to obtain different Wi-Fi signal strengths at the DUT location. All three available power levels (high, medium, low) were used at the router in order to obtain different received signal strength indicator (RSSI) values at the DUT. The RTSA was configured to perform APD measurements. The APD statistical capability of the spectrum analyzer proved to be a valuable choice when measuring non-stationary signals, highlighting fast changes in the received signal [34]. The APD function provided access to the signal power statistics (retrieving mean, peak, and crest factor parameters). Figure 3a presents the power versus time evolution of an upload signal during a 100 ms time interval (802.11 n network, 20 MHz channel bandwidth) and its corresponding APD trace (Figure 3b). 

The APD acquisition time (AQT) was set to 100 ms, which is the beacon time interval of Wi-Fi networks. This corresponds to 6400000 APD symbols, each of 15.62 ns duration, which were considered suitable, given the orthogonal frequency division multiplexing (OFDM) symbol duration of 4 µs used in both Wi-Fi networks (fourth and fifth generations). The other RTSA settings were the following: central frequency (f_c= 2427 MHz/5200 (5190) MHz), resolution bandwidth (RBW = 20 MHz/40 MHz), span = 60 MHz, and AQT = 100 ms.

The total duration of each measurement was 60 s and the field probe was sequentially rotated on all three orthogonal directions Ox, Oy, and Oz. This corresponds to 600 traces of 100 ms each, in each orthogonal direction in space. Mean and peak power values were retrieved by the custom-made Python application. Moreover, average upload/download throughputs (in Mbps) and network RSSI (in dBm) indicated by the Android application were also collected. Only average RSSI and throughput values were retrieved, as the measurements performed by the RTSA could not be perfectly synchronized to the values listed by the Android application. 

Based on the calibration file provided by the manufacturer of the sniffer, the measured power values were transformed into E-field strength values by Equation (1) [36].

(1) $$E\left(V/m\right)=10^{\frac{P+113.2-20lgf}{20}}$$

where *P* is the received power level in dBm provided by the RTSA, *f* represents the channel central frequency in MHz and 113.2 represents the calibration factor provided by the manufacturer in the probe file. 

With E being a spatial vector, the magnitude of the total E field was calculated based on Equation (2) [37].

(2) $$E_{tot}=\sqrt{Ex^{2}+Ey^{2}+Ez^{2}}$$

where *Ex*, *Ey*, and *Ez* represent the averaged (over 60s measurement time) values of the electric field strengths (Root Mean Square—RMS values) on the three orthogonal directions. 

The received power at the sniffer, transformed in E-field strength, was measured in nine situations by repositioning of the Wi-Fi router relative to the phone or by modification of the router emitted power. Practically, each of the nine sets of data consisted in 24 situations [3 probe orientations (Ox, Oy, Oz) × 2 traffic directions (download/upload) × 2 Wi-Fi protocols × 2 bandwidths]. Because both mean and peak APD values were saved to the data file, a total of 432 columns of measured field levels resulted. Each column had 600 rows (one at 0.1 ms) for the analysis. 

The following sources of measurement uncertainty were identified:

(1) RSSI and throughput values were averaged over 60 s (measurement duration). The RSSI value was observed to vary as much as +/−4 dB for the same network configuration and device positioning. Indoor propagation—characterized by multiple reflections, path-loss, scattering, and diffractions—made the real environment extremely unstable.
(2) The positioning of the E field probe (tripod) with respect to DUT was done manually. The positions of both tripods visible in Figure 2 were kept constant. However, differences could occur as the measurement campaign was conducted during several days and the DUT was often removed for charging. The battery level of the DUT was always kept above 50%. A movement of about +/−0.5cm has to be considered.
(3) The three orthogonal directions (Ox, Oy, and Oz) were measured sequentially and the Etot field values were calculated based on Equation (2). This was considered suitable as we estimated similar signal behavior during the total 3-min-long measurement (60 s/each direction).
(4) The spectrum of both 2.4 GHz and 5 GHz bandwidths signals were checked at the beginning of each day in order to identify possible interferences. Based on our interference search, we appreciate that the measurements were conducted in a ‘quiet’ Wi-Fi area. However, short-term interference could not be eliminated. If present, the interferences were more likely to occur in the 2.4 GHz band as it is both more commonly used and has a wider coverage.

### 2.2. Modulation Role on the User’s Exposure Level in IEEE 802.11n and 802.11ac Networks

In 802.11n and 802.11ac links, data rate adaptation was performed by means of changing the modulation and coding scheme (MCS) [38,39]. High order modulations that generally require higher signal to noise ratios (SNR) are used for good channel quality conditions, while more robust modulations like Binary Phase Shift Keying (BPSK) or Quadrature Phase Shift Keying(QPSK) are used in case of poor channel quality. Table 2 presents the MCSs that can be implemented in 802.11n and 802.11ac (20 MHz channel bandwidth) for a single spatial stream (1SS). 

**Figure 4:**
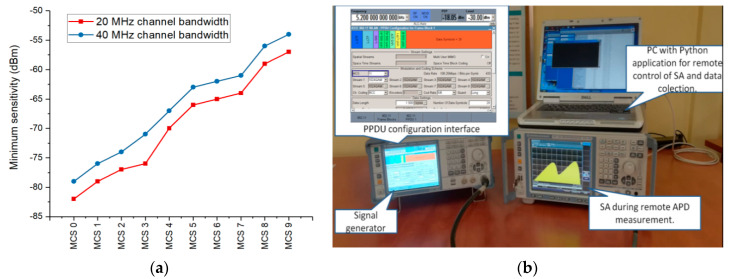
(**a**) Required receive sensitivity of Wi-Fi devices for different modulation and coding schemes; (**b**) Measurement setup used for the controlled experiment.

In Figure 4a, we indicate the minimum sensitivity of a Wi-Fi device required for a specific MCS [39]. One can observe that better sensitivity is required for higher order MCSs. In addition, the 40 MHz channel bandwidth requires a higher sensitivity of the device, as compared to the 20 MHz channel. 

Observations extracted from our deployed networks indicate the MCS as a main influencing factor of the exposure level. Based on these assessments, we designed another controlled experimental setup (Figure 4b), to test how the MCS impacted the exposure of the user. A vector signal generator model R&S SMBV100A was cable-connected to the RF input of the R&S FSVR spectrum analyzer. The R&S SMBV100A generator was equipped with options R&S SMBV-K54 and R&S SMBV-K86 for generating 802.11nand 802.11ac signals, respectively. APD measurements were then performed by applying the same settings as those described in Section 2.1. The main parameters of the generated signals are indicated in Table 3. 

**Figure 5:**
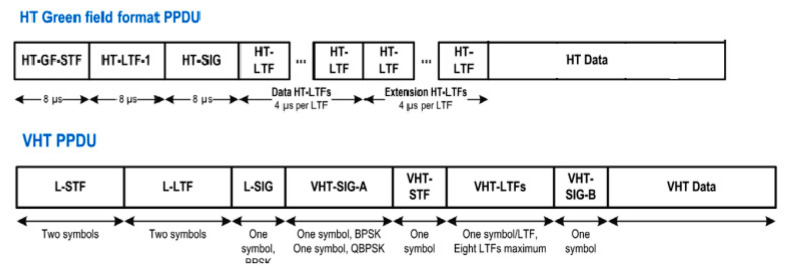
HT Greenfield and VHT physical layer protocol data units in the two generations Wi-Fi standards [41].

The fifth generation of IEEE 802.11 networking standards (802.11ac) is also known as very high throughput (VHT), while the fourth generation (802.11n) is known as high throughput (HT) [40]. In 802.11n, data can be sent by using three different formats of the physical layer protocol data unit (PPDU): non-HT PPDU, HT-mixed format PPDU, and HT- Green Field format PPDU [1,2]. The HT Greenfield format is used in 802.11n network mode solely, while the HT-mixed format PPDU is used for compatibility with previous 802.11a/b/g standards. Given the fact that in the experimental measurements of real Wi-Fi networks, the router was set to 802.11n mode only, Figure 5 describes the structures of the physical layer protocol data unit (PPDU) for HT Green Field format and VHT. 

The physical layer convergence protocol (PLCP) adds a preamble and a header to the transmitted data. The fields of the PLCP preamble and header are visible in Figure 5. Besides this, a PPDU also contains a medium access control (MAC) header and a frame check sequence (FCS) for error detection. 

**Figure 6:**
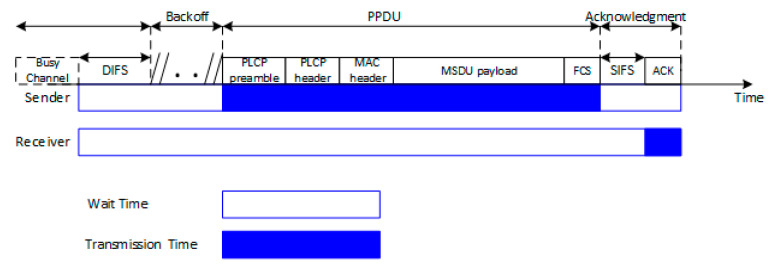
DCF based medium access technique in IEEE 802.11 Wi-Fi networks.

The fundamental medium access control (MAC) technique of the 802.11 Wi-Fi standards is the distributed coordination function (DCF) visible in Figure 6 [42]. DCF employs a carrier-sense multiple access with collision avoidance (CSMA/CA) algorithm in order to sense the wireless medium before transmitting. Accordingly, if a station wishes to transmit, it must listen to the channel status for a DCF interframe space (DIFS) duration [43]. Because multiple stations could be present in the wireless medium, the DCF technique also specifies a random back off time (BO), forcing the station to defer its access to the channel for an extra period. The short inter-frame space (SIFS) is defined as the amount of time required to process the reception of a frame and to respond to it. 

We consider the durations of the DIFS, BO, SIFS, PLCP preamble, PLCP header, MAC header, and FCS constant with respect to MCS index variation within the same standard. The change of the MCS will directly impact the number of the required transmitted symbols for the same amount of data, while the fields listed above remain constant [40]. This implies that different duty cycles will be obtained for different MCSs [30]. In Table 4, we present the physical data rates, the required number of data symbols, and the corresponding duty cycles, as extracted from the control interface of the signal generator for a data length of 1500 bytes.

Theoretically, high MCS schemes should be associated with lower user exposure because of the following main reasons: (1) the use of a higher order MCS provides a smaller duty cycle, thus more idle or wait time; (2) the same amount of data requires fewer numbers of data symbols (transmit time) for a superior MCS than for a lower indexed MCS. Additionally, the duty cycles presented in Table 4 are obtained for continuous transmission, which is not the case of real-life scenarios, when the calculated duty cycles are lower than the theoretical expected maximum values [30]. To this extent, the evaluation of the exposure of the user under different reception conditions (different MCSs), in both controlled setup and real deployed fourth and fifth generation Wi-Fi networks, becomes one of this paper’s objectives. 

**Figure 7:**
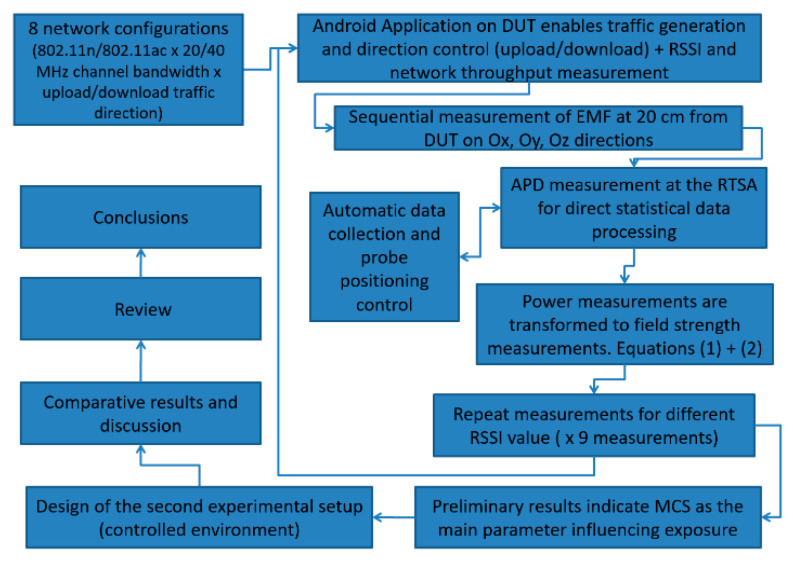
Stages followed for the development of the study.

In Figure 7, we present the flow chart describing the stages followed during the development of this study. 

## 3. Results: Indicators of the Human Exposures in the Configured Experimental Scenarios

**Figure 8:**
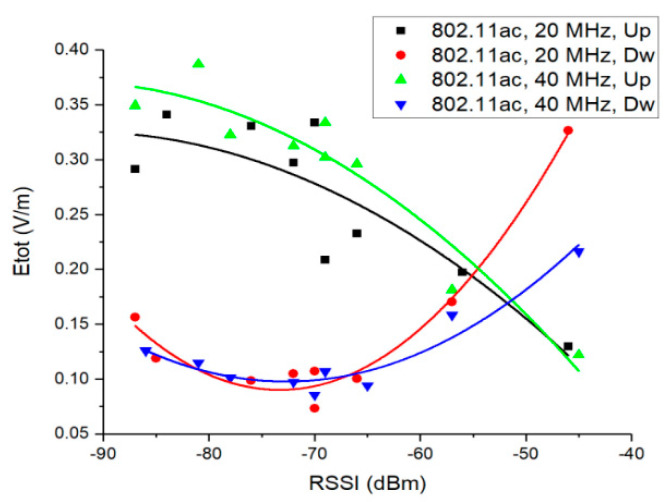
Trend of Etot field levels measured for the 802.11ac networks depending on the DUT RSSI.

Figure 8 presents the mean Etot field strength as a function of the RSSI for 802.11ac networks, uplink and downlink traffic directions. Second order polynomial functions were used for data fitting, as they showed the highest coefficient of determination (R^2^). 

The calculated R² values were 0.75 (802.11ac, 20 MHz, Up), 0.91 (802.11ac, 40 MHz, Up), 0.93 (802.11ac, 40 MHz, Dw), and 0.97 (802.11ac, 20 MHz, Dw). We observe that the total calculated E field strength decreases with the RSSI increase for uplink traffic direction. Thus, experimental measurements performed on real deployed fifth generation network reveal that in the case of the uplink traffic direction, the higher the RSSI (high quality channel propagation conditions), the lower the exposure. This observation is valid for both channel bandwidths, even if it was noticed that the E field values corresponding to the 40 MHz channel are, in general, slightly higher than those measured for the 20 MHz bandwidth (this result converges with the sensitivity graphs presented in Figure 4a). 

One can observe that the trend is different for uplink and downlink traffic directions. To this extent, the total E field values for downlink are the highest for higher levels of RSSI (up to −55 dBm). This is because during the download the router is the main transmitting device, while the DUT operates on reception mode, only transmitting acknowledgement frames (ACK). In the 802.11ac network, following the experimental configuration presented in Section 2.1, in order to obtain a RSSI value of −45 dBm at the DUT, the router was positioned at a 3 m distance from the DUT. Similarly, for a RSSI value of −57 dBm the router was positioned at 6 m from the DUT, in the same room, in line of sight. Practically, for download, the increased values of field level observed for higher RSSIs are due to the smaller distance to the router which is the source of RF field in this situation. 

By increasing the distance between the DUT and the router, RSSI values smaller than −65 dBm were obtained. One can observe that at the tail of the downlink trend lines, the total E field values exhibit the same behavior as in the case of uplink traffic direction. 

**Figure 9:**
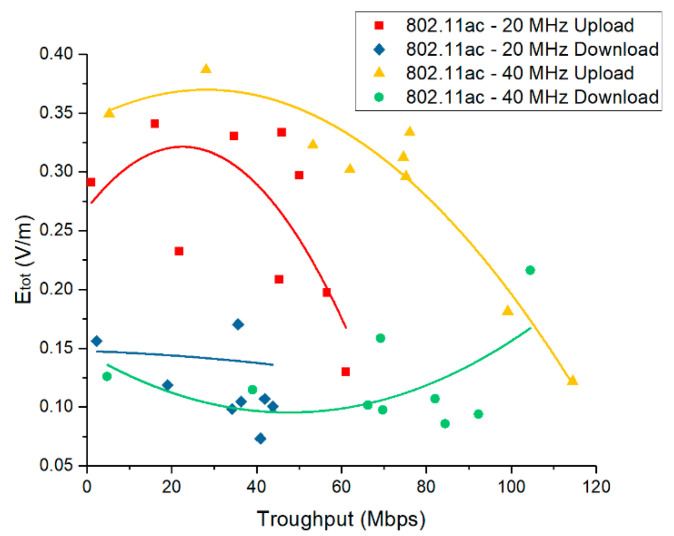
Etot field strengths measured for 802.11ac networks in function of the network throughput.

In Figure 9, we present the total E field strength as a function of the network throughput (measured by means of the Android application). Theoretically, if the link adaptation algorithm would perform at its maximum, a consistent relation should occur between the RSSI values and the network throughput. Our observations show that this might not always be the case in real Wi-Fi networks, as the calculated coefficients of the correlation between the RSSI values and the obtained throughput varied between 0.68 and 0.94. This observation is also supported by [38], which state that there is a significant gap (up to 2.7×) between the throughput with current rate control algorithms and the best achievable throughput. For uplink traffic direction, the highest E field values were obtained for low throughputs, which are generally associated with low RSSI levels. This observation confirms the theoretical expectations stated in Section 2.2. The case is not similar for the downlink because of the proximity issues explained in the previous paragraph. 

**Figure 10:**
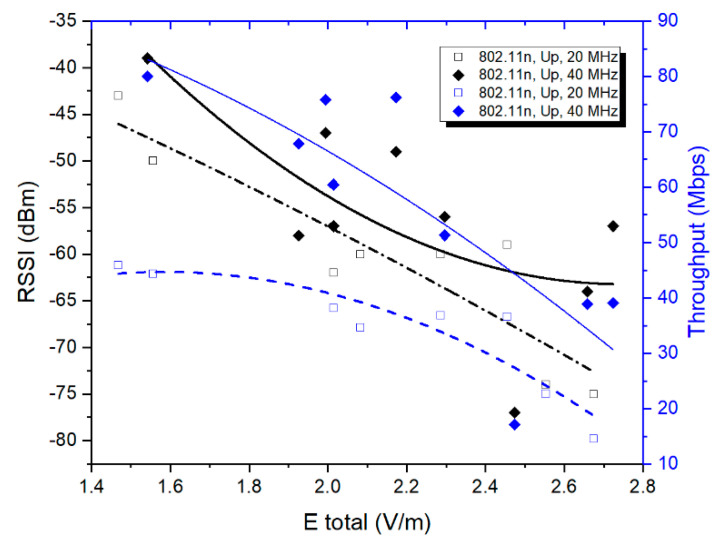
Etot field levels distribution in the 802.11n networks correlated with RSSI and throughput for uplink traffic directions and for two channel bandwidths.

Figure 10 presents the variations of the RSSI and network throughput as a function of the total measured E field strength for the upload traffic direction in two 802.11n networks (20/40 MHz channel bandwidth). One can observe that even if the data were not very well fitted (second order polynomial functions with R² values between 0.52–0.73, which were the best possible), the evolution of the total E field strength was similar to that observed in the 802.11ac network. For the upload traffic direction, lower RSSI conducted to increased user exposure as compared to higher RSSI values. In line with this observation, lower throughputs also conducted to higher measured E field strength values. Some outlier points can be observed. Their presence can be explained by both the sources of measurement uncertainties and by the lack of choosing the proper MCS within the link adaptation algorithm. The differences in field strengths between different RSSI measurement points within the same standard and bandwidth cannot be attributed to different DUT emitted power values, as the 802.11n and 802.ac standards do not specify this degree of freedom for the implemented link adaptation algorithm. Additionally, according to a series of studies, the power control mechanism could not be implemented in 802.11 clients because of the lack of suitable hardware support in wireless cards [43].

**Figure 11:**
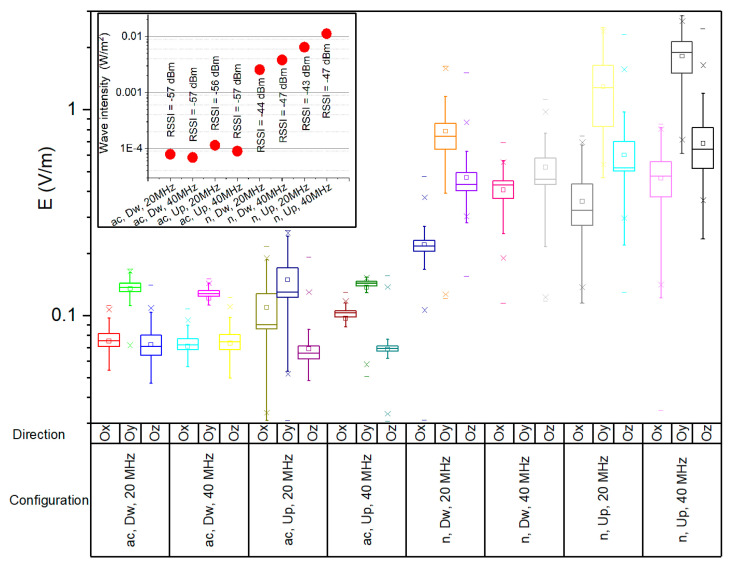
Boxplots of E field strength variability on Ox, Oy and Oz directions in 802.11n and 802.11ac networks (20/40 MHz channel bandwidth) for a single measurement point (for 1 min). Window: Wave intensity (power density) for the single measurement point with corresponding RSSI values.

The variability of the E field strength on all three Cartesian directions in a single measurement point is presented in Figure 11. The boxplots in Figure 11 correspond to a measurement point corresponding to an average RSSI of −45 dBm for 802.11n and to a −57 dBm for 802.11ac standard, respectively. We presented the results in a single situation for simplification purposes; similar distributions were obtained for all the other eight situations. One can observe that the highest field strengths were measured on the Oy direction of the receiving probe, followed by the strengths on Ox (802.11 ac) and Oz (802.11n). Differences between communication protocols occur because the radiation pattern and the parameters of the emission antenna change with frequency. 

The window corner image in Figure 11 presents the calculated wave intensity (red dots) for all eight network configurations in the chosen measurement point. The wave intensity is computed as the time-averaged magnitude of the Poynting vector—i.e., of the power density in air, S_inc_, which in turn is calculated based on Equations (2) and (3) [4,44,45].

(3) $$S_{inc}=\frac{E_{tot}^{2}}{Z}=\frac{E_{x}^{2}+E_{y}^{2}+E_{z}^{2}}{Z}$$

where *S_inc_*represents the power density magnitude, *Etot* represents the total electric field strength as expressed by Equation (2), and *Z* represents the free space impedance (377 Ω). The average is calculated over the 60 s interval of the E-field level tracing.

Both wave intensity and field strength values were higher in the case of 802.11n as compared to those in 802.11ac, regardless of the traffic direction. This could be explained by the following: (a) the higher transmitted power in the 802.11n channel (16 dBm conducted power) than in 802.11ac channel (10 dBm conducted power); (b) higher path loss in the 5GHz frequency bandwidth, which leads to a faster signal attenuation as the distance increases. 

Field strengths for upload traffic were observed to be higher than for download in both 802.11n and 802.11ac networks. In the considered measurement point, the wave intensity of the 802.11n network ranged between 68 µW/m^2^ and 114 µW/m^2^, whereas in the 802.11ac network, it ranged between 2.55 mW/m^2^ and 11.24 mW/m^2^. The corresponding standard deviations were of 19.58 µW/m^2^ for the 802.11ac network and of 3.84 µW/m^2^ for the 802.11n network, respectively. 

**Figure 12:**
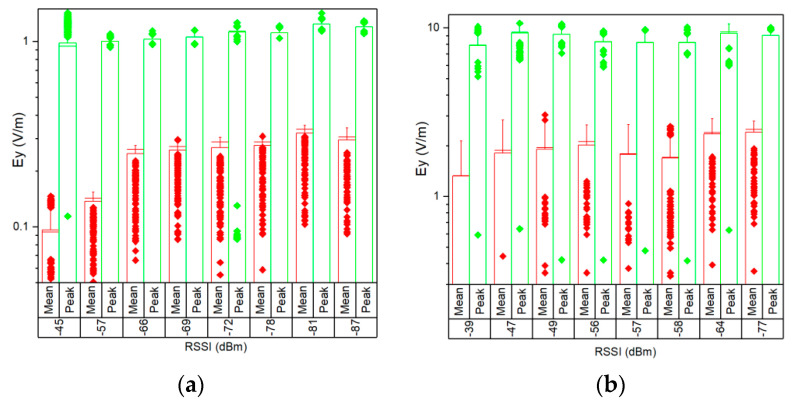
Boxplot distributions of Ey mean (red) and peak (green) field strength values during 1 min versus RSSI, for upload traffic direction, in a 40 MHz channel bandwidth of: (**a**) IEEE 802.11ac; (**b**) IEEE 802.11n networks.

To eliminate the uncertainties due to the sequential measurements (changing directions Ox, Oy, Oz), in the following section the analysis was made using the set of data measured on the Oy direction solely. This was considered suitable for both 802.11n and 802.11ac networks as the Ey field strength made up more than 80% of the total E field strength in all the cases (on average 83.2%). In Figure 12 we present both mean and peak values of the measured E field strengths on the Oy axis, for uplink traffic direction in a 40 MHz channel, for both 802.11n and 802.11ac networks, in eight measurement situations. We chose to represent these values due to the fact that they represent the maximum field values. Again, both the mean and maximum values prove to be higher for the 802.11n network.

For exposure durations below 6 min, the ICNIRP guidelines [4] specify the reference levels in terms of incident energy density. According to [4], in the frequency range 2–6 GHz, incident energy density is calculated based on Equation (4).

(4) $$U_{\mathrm{inc}}=40\;x\;0.36\left[0.05+0.95\sqrt{\left(\frac{t}{360}\right)}\right]$$

where Uinc represents the incident energy density in kJ/m² and t is the time intervals in seconds. 

By applying Equation (4), the calculated maximum permissible incident energy density for a 60-s exposure duration in the frequency range 2–6 GHz is 6.32 kJ/m². The incident energy density is derived as the temporal integration of the incident power density, based on Equation (5) [4].

(5) $$U_{\mathrm{inc}}=\int_{0}^{60s}S_{inc}\left(t\right)dt=\int_{0}^{60s}\frac{E_{x}^{2}\left(t\right)+E_{y}^{2}\left(t\right)+E_{z}^{2}\left(t\right)}{Z}dt$$

By substituting the maximum permissible energy density for a 60-s exposure (6.32 kJ/m²) into Equation (5) and then applying Equation (3), we calculated that it corresponds to a maximum E field level of approximately 200 V/m. The highest E field strength ever recorded within the present measurement campaign was of 11.04 V/m (maximum peak E field value for 802.11 n, upload). This represents only 5% of the ICNIRP general reference level for exposures shorter that 6 min.

**Figure 13:**
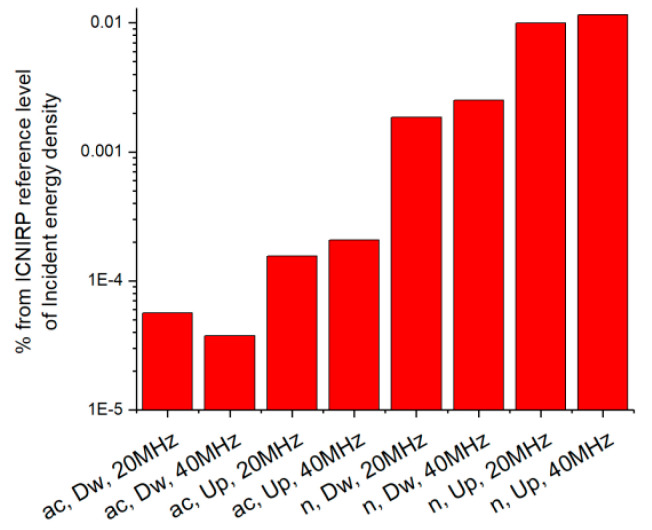
Mean value of incident energy density (averaged over all measurement points) as percentage of the ICNIRP reference level of 6.32 kJ/m².

Figure 13 presents the mean value of the incident energy density, averaged over all the measurement points, as percentage of the ICNIRP reference level for exposure durations shorter than 6 min. One can observe that exposure is much lower than the limit specified in the guidelines, as they reach only 0.0001% (802.11ac) and 0.0064% of the limit (802.11n), respectively. The general observation is that the 40 MHz bandwidth is associated with higher E field strengths than the 20 MHz bandwidth. Basically, for the same RSSI value, a higher bandwidth will decrease the MCS index (increase the duty cycle), resulting in higher mean field strength values. A single exception is visible, namely for the 802.11ac download case, that can be explained by the uneven distribution of the RSSI levels between the measurement scenarios and the aforementioned proximity issue. The average incident energy density values for the 802.11n standard are about 56 times higher than the equivalent values for the 802.11ac standard. However, one should keep in mind that current values were obtained for a single mobile phone model and might not be the same in other cases.

## 4. Modulation: A Significant Indicator of the User’s Exposure in 802.11n and 802.11ac Networks

**Figure 14:**
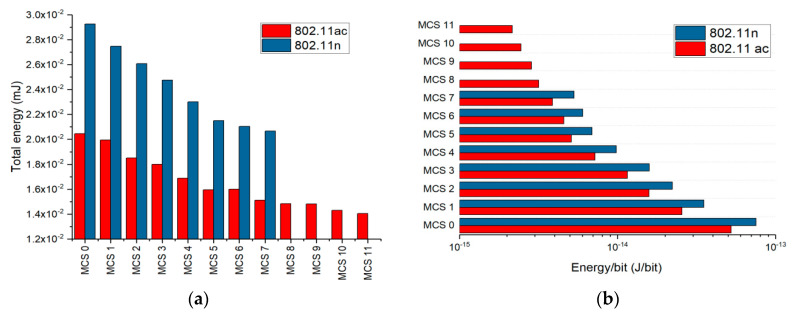
(**a**) Energy used to transmit through a cable different MCSs for IEEE 802.11n and 802.11ac networks during 1-min of data transmission; (**b**) The energy/bit transmitted directly through cable, during 1-min transmission, different MCSs applied in fourth and fifth generation Wi-Fi standards.

In Figure 14a we represented the total calculated energy of the 802.11n and 802.11ac signal within a 60-s measurement time in the case of the controlled setup presented in Section 2.2. The energy in Figure 14 was calculated based on Equation (6) [46].

(6) $$W=\int_{0}^{60s}P\left(t\right)\;dt$$

where *W* is the signal energy accumulated during a 60-s wireless application use, *P*-mean power levels (in mW) measured within the time t = 0.1 s (acquisition time of the APD function as described in Section 2.1). One can notice how the total energy decreases with the MCS index increase for both 802.11n and 802.ac protocols. Practically, higher MCS implies fewer transmitted symbols within a frame, leading to lower duty cycles. Because user experience is evaluated based on the obtained throughput, in Figure 14b we represented the energy required to transmit one bit of information using the available MCS. The result is that the energy per bit increases inversely proportional to the MCS indexing. Ideally, this indicates that lower energies are required to transmit the same amount of information by using high order modulations. 

**Figure 15:**
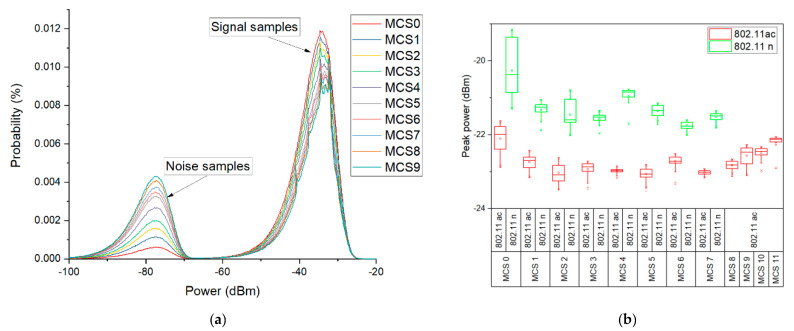
(**a**) Amplitude probability density of signals generated in the 802.11ac standard, at different modulation coding schemes; (**b**) Comparative IEEE 802.11ac and 802.11n standards peak power levels distribution for different MCSs.

Figure 15a shows the APD distribution of the 802.11ac generated signals (controlled experiment). The probability distributions reveal two peaks. One corresponds to the floor noise samples, while the other corresponds to the generated signal samples. As the MCS index raises, the probability of the signal samples to present the received power level of about −35 dBm, decreases. At the same time, the probability of the noise samples raises as higher order modulations exhibit a more stochastic-like nature, with signal values approaching more often the noise floor levels. In Figure 15b, we present the peak power variability of both IEEE 802.11n and 802.11ac signals having different MCSs. We observe that 802.11n peak powers are higher than 802.11ac peak power levels for the same emitted power (see Section 2.2). This is due to the cable loss, which is higher at 5 GHz frequencies as compared to 2.4 GHz frequencies. In addition to this, different variability patterns of the measured peak powers can be observed for different MCSs. The presence of more constellation symbols in the diagram of a modulation will increase the probability for lower amplitude symbols to occur more frequently than in the case of a more robust modulation when only one amplitude level is present. Statistically, this will lead to a decrease in the peak values of the signal, as one can see in Figure 15b. 

**Figure 16:**
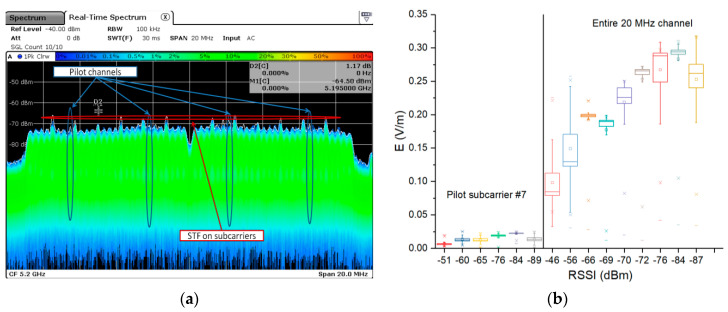
(**a**) Observing the pilot channels over the spectrum of the 802.11 ac signal over a 20 MHz bandwidth; (**b**) E field strength variability—comparison of a pilot channel versus the entire 20 MHz channel over the RSSI dynamic range.

The experimental results obtained in both controlled and real-deployed Wi-Fi networks are presented below by means of the MCS as the main indicator of exposure variability. In Figure 16a, we emphasize the spectrum of the real deployed 802.11ac signal (at 20 MHz bandwidth) captured during file upload (pilots are marked by blue arrows on subcarriers +/− 7 and +/−21). The data transmitted through the pilot channels are always modulated using MCS0 (BPSK) [47]. Accordingly, we measured the E field strength in the pilot subcarrier #7 for several RSSI values and compared it to the E field strength obtained in the entire 20 MHz channel band (only in the case of the Oy direction). It results that the E field strength in the pilot subcarrier is almost flat, whereas the E field strength measured in the entire channel varies significantly. The pilot channel variation was of 0.657 mV/m/dB, whereas the E field strength variation in the entire 20 MHz channel bandwidth was of 10 mV/m/dB. This corresponds to a relative variation of the pilot channel E field over the entire 20 MHz channel E field of 6.57%. The value is appreciated to be within the tolerance defined by the measurement uncertainty. This supports our previous observations in this paper referring to the fact that the E field strength increase is attributed to the MCS index decrease. 

**Figure 17:**
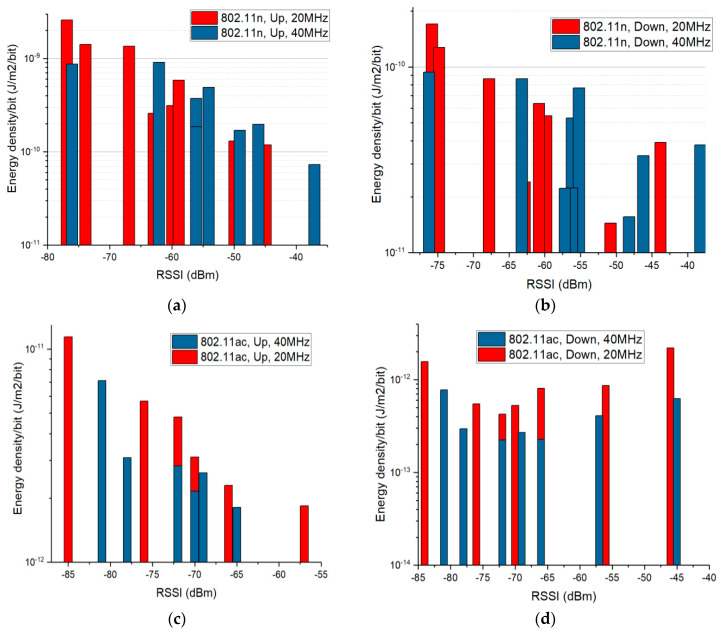
Energy density in air per transmitted bit at a 20 cm distance away from the mobile phone, depending on the RSSI (real deployed Wi-Fi networks), for the cases: (**a**) 802.11n, upload; (**b**) 802.11n, download; (**c**) 802.11ac, upload; (**d**) 802.11ac, download.

Figure 17 presents the calculated energy density per transferred bit in case of the real deployed Wi-Fi networks. The energy density was calculated according to Equation (5)—only Ey values were used; Ex and Ey values were considered null. 

The obtained incident energy density was then divided by the number of bits transmitted within 60 s. The number of bits transmitted within the 1-min interval was calculated by multiplying the average network throughout (measured by means of the Wi-Fi Android application) with the measurement duration (60 s).

For the upload traffic direction (left side of Figure 17), in both 802.11n and 802.11ac networks, we observe that lower energy densities per bit result near the DUT when transmitting under good channel quality conditions (high RSSI), as compared to bad channel conditions (low RSSI). This observation is in line with the results obtained from the controlled setup measurements and with the theoretical hypothesis. In case of download (right side of Figure 17), the effect of the MCS change on the user’s exposure is not evident for high RSSI values, because of the proximity issue explained in the previous subsection. However, the effect is visible for lower RSSI values, when the distance between the E sniffer probe and the wireless router was significantly increased, so that the measured field values could be attributed to a greater extent to the DUT. 

At first sight, the 40 MHz Wi-Fi channels are associated with higher exposure levels than in the case of the 20 MHz Wi-Fi channels (Figure 8, Figure 9 and Figure 10). This observation is not valid if one considers the throughput values during the use of the two bandwidths. To this extent, in Figure 17 one can notice that the energy density per bit in air is lower for the 40 MHz channel bandwidth as compared to the one obtained for the 20 MHz channel bandwidth. This can be explained by the faster rate of throughput increase (theoretically doubled) as compared to the rate of field level increase (1.1–1.2 times higher) when using a wider channel bandwidth. Even if the field value increases when using a wider bandwidth, the user will be exposed for shorter time intervals, because the same amount of data will be transmitted at an increased rate. This results in an overall lower energy density per bit when wider bandwidths are used for the data transmission. 

## 5. Discussion

This paper provides a comparative analysis of a user’s exposure when signals belonging to 802.11n and 802.11ac communication networks are the source of radiation. The analysis in Section 3 is based on the following four physical quantities used in the evaluation: the total electric field strength in a point in air (reconstructed from the separate measurements of the three spatial components of the vector field)—Figure 8, Figure 9 and Figure 10; the electric field strength on the dominant polarization direction (E_y_)—Figure 12; the incident power density/wave intensity)—Figure 11; and the incident energy density—Figure 13. 

Firstly, none of the measured values (electric field strength, incident wave intensity or incident energy density) exceeds the ICNIRP reference levels for local exposure. However, EMF was measured in case of a network with a single Wi-Fi user and exposure is expected to increase when additional Wi-Fi devices will be connected. Even so, preliminary results do not indicate a possible health hazard as defined by the standards [3,4]. The measured field values are convergent with several previous findings [22,23,27], if differences in research design are considered.

Secondly, the exposure is higher in the IEEE 802.11n network as compared to the 802.11ac network. This assertion is valid for the measured DUT model that presents a lower conducted power in the 5 GHz bandwidth than in the 2.4 GHz bandwidth. The fastest fading in the case of higher frequency (5 GHz) signals also sustains this statement. 

The conducted power and the path loss are not the only variables influencing the exposure level in this case. The gain of the phone’s Wi-Fi antenna in the direction of measurement is another influencing factor. We know that the total gain is indicated by the product of the antenna efficiency and its directivity. Wi-Fi antenna efficiencies for handheld mobile devices are typically on the order of −6 dB to −2 dB [48]. To enhance the generality of our conclusion, we would like to add the following:

(a) Higher E field strength values were also recorded for 802.11n network as compared to those obtained in the case of the 802.11ac network in some of our previous findings, as reported in [49], when computer clients were used instead.
(b) Similar findings are expected for other mobile devices that exhibit higher conducted power in the 2.4 GHz Wi-Fi frequency band as compared to that in the 5 GHz Wi-Fi frequency band (e.g., found by the authors in the FCC database of phones: Huawei P30 Lite, LG G6, HTC One).
(c) Additional research supporting this conclusion was identified. If we consider that the radiated power is directly proportional to the power consumption of the mobile handset, then [50] indicates (in Figure 4b) that the power consumption was higher for the 802.11n standard as compared to the one in the 802.11ac standard considering the same bandwidth size for a Samsung smartphone model. If we also take into consideration the path loss, we can estimate that the radiated field will be lower for 5 GHz frequency range as compared to the 2.4 GHz frequency range.

Thirdly, exposure depends on the direction of the data exchange. The upload traffic conducts to a higher exposure level than the download traffic at the same distance from the source of radiation. This observation is also sustained by the findings presented in [31,32].

In addition to the distance from the radiation source and the emitted power, user exposure is dependent on the received signal quality (Wi-Fi coverage quality). For the upload, the worse the signal quality, the higher the exposure is. For the download, the distance from the wireless router seemed to be a more powerful factor than the RSSI (applied MCS). The influence of the MCS was visible only for low RSSI levels when considerable distance was set between the wireless router and the DUT.

Within the investigated RSSI range (55 dB and 42 dB, respectively), the exposure was found to vary with 9.13 V/m (maximum – minimum value) in the IEEE 802.11n networks and with 1.32 V/m in the 802.11ac network, respectively. The standard deviation of all E field strength values measured across all the RSSI range was of maximum 2.78 V/m for the 802.11n network and of 0.36 V/m for the 802.11ac network. The higher variability of the measured values for the 802.11n network can be explained by several factors. First, it is the highest conducted power in the antenna of the DUT. Second, the wavelength is also higher in the case of a 2.4 GHz frequency band. The measurement point (20 cm distance away from the DUT) was thus localized closer to the near field region, where higher variability of the EMF is expected. Third, the 2.4 GHz band is much more crowded than the 5 GHz band, thus it is more susceptible to external interference. 

The network throughput was found to be inversely proportional to the electric field strength for the upload (DUT emission) in both the 802.11n and the 802.11ac networks. The observation is consistent with the results presented in [31], but contradicts the results presented in [27]. However, in [27] the 802.11b and the 802.11g Wi-Fi networks were analyzed. Until further research is carried out, the present findings remain valid only for the 802.11n and the 802.11ac Wi-Fi networks. 

In Section 4, the MCS role on the exposure outcome is investigated. The controlled setup experiment indicated clear evidence that the lower order MCSs are associated with higher exposure. Differences were observed in terms of total signal energy (mJ) and they were even more prominent when the energy per bit indicator was used (mJ/bit). For radiated signals we used the energy density per bit to highlight the differences in exposure profiles as retrieved from real deployed Wi-Fi networks. A 1× $10^{-9}$ mJ/m²/bit variation was found for the upload 802.11n link and $9\times 10^{-12}$ mJ/m²/bit for the upload 802.11ac link, respectively. These imply that uploading 10 Mb of data in a 802.11n network will result in an incident energy density of 0.097 mJ/m² for a RSSI of −45 dBm and of 0.88 mJ/m² for a RSSI of −78 dBm, respectively. Far below the ICNIRP reference levels [4], this represents an increase of incident energy density of 9.07 mJ/m² within an RSSI span of 33 dB that could be significant for establishing non-thermal health effects of Wi-Fi users. 

## 6. Conclusions

Present results offer an original insight into the exposure assessment of users of the fourth and fifth generation Wi-Fi networks. 

Realistic exposure assessment in Wi-Fi networks was consistently improved by applying duty cycle-based weighting methods [30,31]. However, the methods may still not be able to completely follow the stochastic-like behavior of real deployed Wi-Fi signals. 

By means of the current approach we have demonstrated that in the case of IEEE 802.11n and 802.11ac communication standards, the user’s exposure is dependent on the used MCS by means of a theoretical analysis and an experimental validation in both controlled and uncontrolled sessions. Because of the direct relation that exists between the RSSI and the MCS (which is also connected to the duty cycle of transmission), the current findings might represent a steppingstone towards the development of a useful mobile application. The mobile application may provide the user exposure based on easily accessible parameters (RSSI monitoring, Wi-Fi upload/download data retrieved from data monitoring applications). In Wi-Fi networks, an MCS-based exposure assessment tool will certainly prove its agility in profiling the user exposure. This provides valuable information for long term epidemiological studies related to EMF exposure. The design, testing, and validation of such an application constitute one of our future research directions.

In addition, the use of the energy density alone, as referenced in the ICNIRP guidelines, may not be applicable for comparing dosimetric situations that imply the use of different modulation types. This is because in real life situations the users are interested in improving the quality of the radio communication link in terms of data rates and wait-times. Different modulations may exhibit similar energy densities within the same time span, but the amount of transferred data can vary significantly. Therefore, in the present paper we have introduced and used for the first time, to our knowledge, the metric called incident energy density per unit of transmitted information (bit) and proved its superiority in highlighting the differences between electromagnetic exposure profiles. 

## Figures and Tables

**Table 1:** Wi-Fi network configurations.

Network Number	IEEE Protocol	Channel Number	Central Frequency (MHz)	Channel Bandwidth (MHz)
1	802.11n	4	2427	20
2	802.11n	4	2427	40
3	802.11ac	40	5200	20
4	802.11ac	38	5190	40

**Table 2:** Modulation and coding schemes used in 1SS Wi-Fi networks.

MCS Index	IEEE 802.11n Protocol			IEEE 802.11ac Protocol		
	Modulation	Coding Rate	Bits/Symbol	Modulation	Coding Rate	Bits/Symbol
MCS 0	BPSK	1/2	26	BPSK	1/2	26
MCS 1	QPSK	1/2	52	QPSK	1/2	52
MCS 2	QPSK	3/4	78	QPSK	3/4	78
MCS 3	16-QAM	1/2	104	16-QAM	1/2	104
MCS 4	16-QAM	3/4	156	16-QAM	3/4	156
MCS 5	64-QAM	2/3	208	64-QAM	2/3	208
MCS 6	64-QAM	3/4	234	64-QAM	3/4	234
MCS 7	64-QAM	5/6	260	64-QAM	5/6	260
MCS 8				256-QAM	3/4	312
MCS 9				256-QAM	5/6	346
MCS 10 *				1024-QAM	3/4	390
MCS 11 *				1025-QAM	5/6	433

* MCS-10 and MCS-11 (1024-QAM) are supported by several 802.11ac chipsets [40].

**Table 3:** Generated signals parameters for the controlled experiment.

Parameter	Value	
	IEEE 802.11 n Protocol	IEEE 802.11 ac Protocol
RF frequency	2.427 GHz (channel 4)	5.2 GHz (channel 40)
Channel bandwidth	20 MHz	20 MHz
Level	−30 dBm	−30 dBm
Spatial streams	1	1
Frames number	500	500
Data source	Predefined resource (P9)	Predefined resource (P9)
MCSindex	0–7	0–11
Mode	HT-20MHz (Greenfield)	VHT-20 MHz
Data length	1500 bytes	1500 bytes
MAC Header	Enabled	Enabled

**Table 4:** Number of data symbols and duty cycles for various MCSs in 802.11n and 802.11acnetworks.

MCS Index	IEEE 802.11 n Protocol			IEEE 802.11 ac Protocol		
	Data Rate (Mbps)	Number of Data Symbols	Duty Cycle (%)	Data Rate (Mbps)	Number of Data Symbols	Duty Cycle (%)
MCS 0	6.5	469	0.95	6.5	469	0.95
MCS 1	13	235	0.906	13	235	0.907
MCS 2	19.5	157	0.867	19.5	157	0.869
MCS 3	26	118	0.832	26	118	0.836
MCS 4	39	79	0.772	39	79	0.78
MCS 5	52	59	0.722	52	59	0.734
MCS 6	58.2	53	0.702	58.2	53	0.715
MCS 7	65	47	0.679	65	47	0.695
MCS 8	-	-	-	78	40	0.66
MCS 9	-	-	-	86.5	36	0.647
MCS 10	-	-	-	97.5	32	0.626
MCS 11	-	-	-	108.25	29	0.609
